# Phytochemical Analysis, Antioxidant Potential, and Cytotoxicity Evaluation of Traditionally Used *Artemisia absinthium* L. (Wormwood) Growing in the Central Region of Saudi Arabia

**DOI:** 10.3390/plants11081028

**Published:** 2022-04-09

**Authors:** Hamdoon A. Mohammed

**Affiliations:** 1 Department of Medicinal Chemistry and Pharmacognosy, College of Pharmacy, Qassim University, Buraydah 51452, Saudi Arabia; ham.mohammed@qu.edu.sa; Tel.: +966-566-176-074; 2 Department of Pharmacognosy, Faculty of Pharmacy, Al-Azhar University, Cairo 11371, Egypt

**Keywords:** *Artemisia absinthium*, davanones, antioxidant activity, cytotoxic effect, GC-MS, GC-FID, essential oil quality

## Abstract

*Artemisia absinthium*, a plant distributed worldwide, has been reported for its numerous traditional uses, and its phytoconstituents have been investigated in several previous publications. The current study was designed to investigate the chemistry and quality; i.e., the antioxidant and cytotoxic activities, of *A. absinthium* volatile oil from plant species growing in the central area of Saudi Arabia compared to reported data for the plant growing in other parts of the world. Gas chromatography–mass spectrometry (GC-MS) and gas chromatography with flame ionization detector (GC-FID) spectroscopic analyses, in addition to in vitro antioxidant and cytotoxic assays, were conducted to fulfill the aims, and integrated the study’s conclusion. A total of 34 compounds representing 99.98% of the essential oil of the plant were identified; among them, cis-davanone was found at the highest concentration (52.51%) compared to the other constituents. In addition, α-gurjunene (7.15%), chamazulene (3.38%), camphene (3.27), γ-eudesmol (2.49%), pinocarvone (2.18%), and ocimenone (2.03%) were also identified as major constituents of the plant’s essential oil. The total percentage of davanones (53%) was the highest percentage found in the plant species growing elsewhere in the world. The antioxidant assays; i.e., the total antioxidant capacity (TAC), ferric-reducing antioxidant power (FRAP), and 2,2-diphenyl-1-picrylhydrazyl-scavenging activity (DPPH-SA), evidenced the potential in vitro antioxidant activity of the *A. absinthium* essential oil, with 35.59, 10.54, and 24.00 mg Trolox equivalent per gram of the essential oil. In addition, the metal-cheating activity (MCA) of the essential oil was measured at 29.87 mg ethylenediaminetetraacetic acid (EDTA) equivalent per gram of the essential oil. Moreover, a limited cytotoxic effect of the essential oil against all tested cell lines was observed, which might be considered as an indicator of the safety of *A. absinthium* as a worldwide edible plant. In conclusion, the study confirmed the variations in the *A. absinthium* essential oil constituents in response to the environmental conditions. The study also highlighted the potential health benefits of the plant’s essential oil as an antioxidant agent.

## 1. Introduction

Aromatic plants have been utilized for human use as food, flavoring agents, and medicaments for the treatment of various diseases. They are also considered as chief materials in many industries; e.g., in pharmaceutical preparation and perfume preparation. Aromatic-therapy with plants and their derived volatile oils has been given a high economic value around the world [1]. Several factors affecting the quality and diversity of volatile oils in particular aromatic plants, have been reported [2,3]. Among them, the major factor is the variation in environmental conditions in the area where the plant grows [4,5,6]. Therefore, the production, chemistry, and quality of the volatile oil of a particular plant could be significantly affected by temperature, soil contents, drought, and the environmental conditions of marshes [2,3,5,7,8].

The *Artemisia* is a genus of more than 500 species of aromatic plants belonging to the family Asteraceae [9]. The plants belonging to the genus *Artemisia* are widely distributed in the world, and are greatly used in folklore medicinal applications and complementary medicine systems in several countries [6]. The genus *Artemisia* also includes several plants used in modern medicine for the treatment of inflammatory diseases, gastrointestinal disorders, fever, malaria, hepatitis, and cancer [10,11,12]. The plants of this genus also used for treatment of infectious diseases of fungal, bacterial and viral origins [9]. The activities of plants related to this genus are partially attributed to their volatile oil contents [9,13,14], in addition to the presence of other secondary metabolites; e.g., bitter principles, flavonoids, and alkaloids [15,16,17]. Among the important *Artemisia* species is *Artemisia absinthium*, or wormwood, a plant that grows worldwide in different climatic regions [18,19,20,21]. *A. absinthium* has been also reported for its spreading in Europe and Siberia [9,22,23]. In addition, this plant is widely distributed in the Mediterranean region and Gulf countries [24,25,26,27]. Therefore, several local names have been given to the plant based on the growing location; e.g., the plant is called majtari, yang ai, wermut, and afsanteen in India, China, Germany, and Egypt, respectively, in addition to the English name, wormwood [28]. Several compounds have been identified as major volatile constituents of the plant, and different chemotypes were reported for the plant according to the growing region and the diversity of the growing conditions (Table 1). In addition to the volatile constituents of *A. absinthium*, the plant also contains phenolic acids, flavonoids, isoflavonoids, and tannins [29,30,31]. The clinically importance sesquiterpene lactone bitter principle, artemisinin, also has been identified in the plant [32].

The worldwide distribution of *A. absinthium* also accompanies several traditional uses for the plant. For instance, *A. absinthium* has been identified as one of the medicinal plants used by the ancient Egyptians, and was found to be one out of the 328 ingredients written in the famous Ebers Papyrus in 1550 BC [45]. The plant also is used traditionally in the treatment of cancer, hepatitis, jaundice, wounds, GIT disorders (dyspepsia, flatulence, and colic), depression, and gout; and as an insect repellent and anthelmintic [31,32,46,47,48]. From a pharmacologic perspective, antimalarial, antiprotozoal, antimicrobial, anticancer, antioxidant, and hepatoprotective activities have been reported for *A. absinthium* extracts and pure isolated compounds from the plant [49,50,51,52,53,54].

The current study investigated a phytochemical analysis of *A. absinthium* volatile oils for the plant species growing in the central area of Saudi Arabia. In addition, the effects of environmental conditions on the volatile constituents of the plant were also discussed by comparing the results of the current analysis with the reported volatile constituents of the plant. The study also provided data on the antioxidant and cytotoxic effects of the volatile oil of *A. absinthium*.

## 2. Results and Discussion

### 2.1. Essential Oil Production and Constituents of A. absinthium

*A. absinthium* is one of the most widely distributed and commonly used aromatic plants in the world. Its volatile constituents have been investigated and described for the plant’s species growing in different locations and countries; e.g., Egypt, Tunisia, Algeria, Spain, Germany, Siberia, Lithuania, and Turkey. However, these different countries varied in their environmental conditions, which affected the plant’s volatile oil constituents and production (Table 1), and subsequently the quality and biological activities of the oil. In the current study, the volatile oil of the *A. absinthium* species growing in the central region of Saudi Arabia was distilled and analyzed using GC-MS and GC-FID spectrometry (GC-chromatogram is available in the supplementary file). The result obtained for the plant’s volatile oil production (0.94 ± 0.03% *w*/*w*) was slightly higher than the reported percentage of volatile oil production obtained from same species growing in the southwest region of Saudi Arabia (0.83% *v*/*w*), at a distance of more than 1000 km [24,33] away from the Qassim region. This large distance between Qassim on one side and Al-Baha and Jazan on the other side was accompanied by distinct variations in the environmental conditions of these locations. In March, for the plants collected in the current study and in the reported study [24], temperatures of 7–18 °C and 17–27 °C and humidity levels of 83% and 25% were recorded for the Jazan and Qassim regions, respectively. In addition, Qassim is characterized by flat land, while Jazan and Al-Baha are mostly mountainous [55]. Moreover, the soil contents also vary, as Qassim is mostly characterized by the presence of a high salt content in the soil. The environmental conditions’ effects on the plant constituents were clearly demonstrated by comparing the results obtained using the GC analysis of the volatile oil sample of *A. absinthium* growing in central Saudi Arabia (Table 2) with the reported results of the plant’s volatile constituents from other areas of Saudi Arabia and the world.

The GC-MS and GC-FID analyses revealed the presence of 34 volatile constituents in the distilled essential oil of *A. absinthium* growing in central Saudi Arabia. Among all the identified compounds, cis-davanone was found at the highest percentage (52.51%) of the total volatile constituents of the plant. Davanone was recorded as a common chemotypic constituent of other *Artemisia* species; e.g., davanone was found to be 57.32% of the essential oil of *A. chamaemelifolia* [56]. In addition, *Artemisia indica* was described as a source of davanone, and the percentage of davanone in the plant’s essential oil was found to be 30.80% [57]. Davanone has been found in high concentrations in *Artemisia aucheri* (~23%) [58], *Artemisia fragrans* (16.1%) [59], and *Artemisia pallens* (53.0 %) [60].

Furthermore, α-gurjunene (7.15%), chamazulene (3.38%), camphene (3.27), γ-eudesmol (2.49%), pinocarvone (2.18%), and ocimenone (2.03%) were also identified as major constituents of the plant’s essential oil. Moreover, 10 compounds; i.e., α-pinene, β-ocimene, cis-linalool oxide, trans-thujone, bornyl acetate, ethyl hydrocinnamate, γ-curcumene, davanone, cubenol, and geranyl tiglate were identified at concentrations below 2% and above 1%. All other constituents (17 compounds) were identified at concentrations below 1% (Table 2). The identified constituents were mainly categorized as oxygenated sesquiterpenes with a total percentage of 63.61%, followed by nonoxygenated sesquiterpenes, which were calculated as 14.69% of the total identified compounds. The monoterpenes were calculated as 20.86%, distributed as 9.82% of the nonoxygenated monoterpenes and 11.04% of the oxygenated monoterpenes.

**Figure 1:**
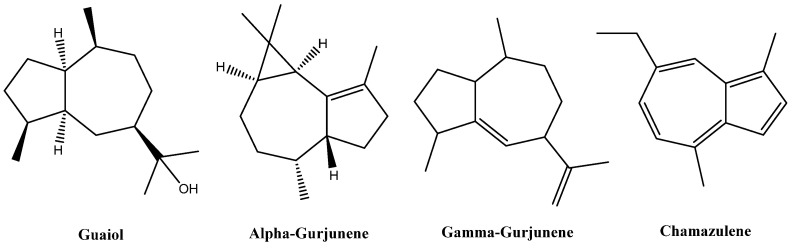
Chemical structure of the azulenes in *A. absinthium* essential oil.

The chemotypic variations among the *A. absinthium* plants growing in different locations might be clearly determined by comparing the major constituent of the plant in the current study; i.e., cis-davanone, which was also reported to be the major constituent of the plant samples collected from other regions of Saudi Arabia [24,33] compared to the major constituents; e.g., chamazulene, cineole, and trans-sabinyl acetate, reported in the plant samples growing in other regions of the world [38,39,40,42]. In addition, the colors of the *A. absinthium* essential oils from different regions were varied, and seemed be associated with the major constituents of the plant’s essential oils [23]. For instance, the essential oils obtained from *A. absinthium* growing in Tunisia, Algeria, China, and southern Saudi Arabia were reported to be dark blue in color, which was attributed to the presence of chamazulene (approximately ≥8%) as one of the major constituents of the plants’ essential oils [24,34,35,61,62,63]. In addition, essential oils obtained from Lithuanian *A. absinthium* were described in the literature as dark brown in color, with major constituents including trans-sabinyl acetate, β-pinene, trans-thujone, cis-thujone, and myrcene; however, chamazulene did not exceed a concentration of 1.5% [37,38]. Also, a yellow-colored essential oil was obtained from *A. absinthium* growing in Tajikistan, in which chamazulene was detected as one of the minor constituents of the essential oil [40]. The essential oil of *A. absinthium* obtained in this study was yellow in color, which might be attributed to the lower concentration of chamazulene (3.38%) compared to the reported concentration of the compound in the blue *A. absinthium* essential oil. The yellow color of the essential oil in the current study might also be attributed to the presence of other major constituents; e.g., cis-davanone (52.51%), α-gurjunene (7.15%), and camphene (3.27%). When compared to the current analysis of the plant’s essential oil, the blue-colored chamazulene was also identified at a higher concentration in the volatile oil of *A. absinthium* growing in the southern area of Saudi Arabia, at a concentration of 8.2% versus 5.04% for the species of the plant growing in Jazan and AL-Baha, respectively [24,33]. This significant variation in the chamazulene concentration among the Saudi Arabian *A. absinthium* species seems to be a direct reason for the color variation (blue to yellow), and also might be associated with a variation in the biological activities of the plant’s essential oil. However, identification of semisaturated azulenes; i.e., α-gurjunene (7.15%), γ-gurjunene (0.86%), and guaiol (0.49%), in addition to 3.38% of the chamazulene, in the current GC-MS and GC-FID analyses was an indication of the liability of the plant to biosynthesize azulenes as its major sesquiterpene constituents (Table 2). The representation of these azulene derivatives could be also indicative of part of the possible chemical bioconversion in the plant’s essential oil constituents (Figure 1).

### 2.2. Antioxidant Activity of A. absinthium Essential Oil

Four different in vitro assays were conducted to evaluate the antioxidant potency of the *A. absinthium* essential oil. These assays measured the reducing effect (i.e., TAC and FRAP), scavenging ability (i.e., DPPH), and metal-chelating activity (i.e., MCA) of the essential oil of the plant. The measurements were conducted in a comparable manner to the standard antioxidant compound, Trolox, and standard metal-chelating agent, EDTA. Therefore, the results were expressed as Trolox equivalents in the TAC, DPPH, and FRAP; however, the result of the MCA was expressed as the EDTA equivalent (Table 3).

The antioxidant activities of *A. absinthium* essential oils have been recorded several times in the literature [35,43,44,64,65,66,67]. The methods that were used in most of the reports were different from the methods used in this study; therefore, comparing current antioxidant results to the reported data might be conflicting. However, few studies reported similar antioxidant methods. For instance, a phosphomolybdenum assay (TAC) was conducted using standard calibration curves obtained for α-tocopherol compared to the essential oil obtained from *A. absinthium* growing in Turkey [68]. The result was measured as 2.89 ± 0.16 mM α-tocopherol equivalent, compared to 35.59 ± 1.86 for the total antioxidant capacity of the *A. absinthium* essential oil in the current work. The ferric-reducing antioxidant capacity (FRAP) of the essential oil of *A. absinthium* growing in Iran was calculated as 10.67 ± 0.45 mg/g gallic acid equivalent, which was significantly lower than the current result for FRAP, at 24.00 ± 0.13 mg/g of the essential oil as the Trolox equivalent [44].

The overall results represented in Table 3 revealed the potential antioxidant power of the volatile oil of *A. absinthium* growing in the central area of Saudi Arabia, and also indicated that the environmental conditions of the area where the aromatic plants grew represented a primary factor affecting the chemistry and quality of the essential oils obtained from these plants. The current higher antioxidant activity of the essential oil of *A. absinthium* could be attributed to the presence of a high percentage of davanones, which were found collectively to be more than 53%. Plants rich in davanones have been reported for their interesting antioxidant activities; specifically, scavenging free radicals [58,59]. The higher percentages of oxygenated sesquiterpenes in the essential oil of the plant could be also a reason for the higher antioxidant activity in the current results. The oxygenated sesquiterpenes, guaiol (0.49%) and globulol (1.38%), have shown antioxidant power against thiobarbituric-acid-reactive species [69]. In addition, the presence of ocimene (1.65%); 1, 8-cineole (0.87%); α-terpinene (2.68%); cis-linalool oxide (1.55%); camphene (3.27%); and α-thujene (0.35%), which have been reported for their antioxidant effects as scavengers of the thiobarbituric-acid-reactive species and linoleic acid peroxidation [69], could also affect the antioxidant activity of *A. absinthium*.

### 2.3. Cytotoxic Activity of A. absinthium Essential Oil

**Figure 2:**
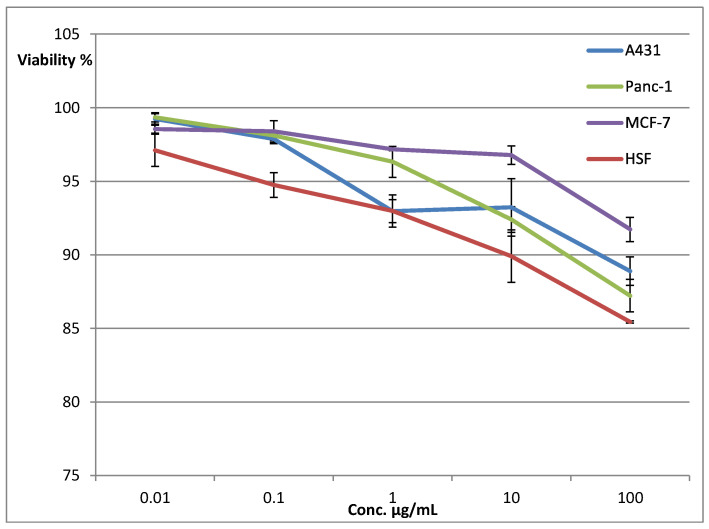
Viability percentages of the cancerous cells A431 (human epidermoid skin carcinoma), Panc-1 (pancreatic cancer), MCF-7 (breast adenocarcinoma) and the normal HSF fibroblast cells (human skin fibroblast cells) treated with different concentrations of the *A. absinthium* essential oil.

The cytotoxic effects of the *A. absinthium* essential oil were evaluated at five different concentrations (in a range of 0.01 to 100 µg/mL) against three cancer cell lines; i.e., A-431, MCF7, and PANC-1, as well as one normal cell line; i.e., HSF, using the standard SRB (sulforhodamine B) assay (Table 4, Figure 2). An SRB assay is used to measure cell masses and proliferation in an acidic medium. The SRB binds quantitatively to cell proteins, and can be extracted using a basic solvent [70]. The blank absorbances were measured (0.02 to 0.04 A.U.) and were subtracted from the absorbances of the tests. The negative-control absorbances were also measured as 1.6373, 1.344, 2.287, and 2.816 for the MCF-7, Panc-1, A-341, and HSF cell lines, respectively (the raw data are available in the supplementary file, Table S1–S4). The results presented in Table 4 revealed that the *A. absinthium* essential oil showed negligible cytotoxicity against all the tested cancer cell lines, and did not exceed 8.27%, 12.77%, and 11.10% of the activity against the three cancer cell lines MCF7, PANC-1, and A-431, respectively. The weak cytotoxic effect of the *A. absinthium* essential oil was clearly demonstrated when it was compared with the results obtained from the positive control, doxorubicin (DOX). At all concentrations;, i.e., 0.01–100 µg/mL, DOX’s antiproliferative effects against all cell lines were much stronger compared to the *A. absinthium* essential oil. In addition, doxorubicin at the highest concentration (100 µg/mL) showed increased cytotoxic activity by 8.8×, 6.3×, and 5.9× times compared to the *A. absinthium* essential oil against the MCF-7, Panc-1, and A-431 cancer cell lines, respectively (Table 4).

It was noteworthy that the susceptibility of the HSF normal cell line to the essential oil of *A. absinthium* was higher than that of the cancer cell lines at all concentrations (Figure 2). The higher sensitivity of the HSF cell line to the *A. absinthium* essential oil compared to the other cancer cell lines was also clearly demonstrated as shown in Figure 2 and Table 4, which show a significantly higher cytotoxic effect of the plant’s essential oil against the HSF cells (14.56%) compared to the MCF-7, Panc-1, and A-431 cell lines. The overall lower cytotoxic effect of the *A. absinthium* essential oil might be considered to signal a higher safety of this plant and its essential oil, primarily based on this plant being used as food without any record of toxicity or side effects [71,72]. A cytotoxic effect of *A. absinthium* essential oil from different regions of the world has been reported. The essential oil of the plant growing in Lithuania [73] showed a cytotoxic effect on brine shrimp, with IC_50_ values in the range of 15–31 µg/mL; however, this toxicity could be attributed to the presence of a higher concentration of thujones, which are well-known cytotoxic monoterpenes [72], at a concentration of 12.3% in the Lithuanian plant [73]. However, the total percentage of trans-thujone, the only thujone found in the plant’s essential oil, was calculated in current study as 1.14% of the plant’s total essential oil constituents.

## 3. Materials and Methods

### 3.1. Plant Collection and Volatile Oil Preparation

The plant materials as aerial parts (obtained from ≈100 plants) were collected in March 2020 from private gardens in Qassim, Saudi Arabia. The plant materials were identified as *Artemisia absinthium* L. by the botanist at the Department of Plant Protection and Production, College of Agriculture, Qassim University. The plant samples were kept at the Department of Medicinal Chemistry and Pharmacognosy, College of Pharmacy, Qassim University. The plant materials were dried under shade in the conditions of the room for 10 days and ground to powdered form. The volatile oil was distilled from the ground plant’s aerial parts using a Clevenger apparatus in which 450 g of the plant materials were divided into three equal parts (150 g each). Each part was suspended separately in 1 L of distilled water in a conical flask and allowed to boil for 3 h over a heating mantle. The distilled oils were separated from the aqueous layer using a separating funnel, and then dried over anhydrous sodium sulfate powder. The total yield of the oil was calculated as the mean of three distillation processes (0.94 ± 0.03% *w*/*w*) of the dried aerial-part materials of the plant.

### 3.2. Gas Chromatography–Mass Spectroscopy Analysis of the Volatile Oil

An Agilent 8890 GC system attached to a PAL RTC 120 autosampler equipped with a HP-5 capillary column (30 m, 250 µm i.d., 0.25 µm film thickness) was used to separate and analyze the essential-oil components of the *A. absinthium.* The system was also equipped with an Agilent 9977B GC/MSD mass spectrometer (Agilent technology, Santa Clara, CA, USA). The initial isothermal column temperature was kept at 50 °C for 2 min. The column temperature was automatically adjusted upward until reaching 220 °C in intervals of 5 °C/min. Finally, the column temperature was elevated to 280 °C (at rate of 10 °C/min) and remained at 280 °C for 10 min (isothermal). In addition, the temperature of the injector was kept constant at 230 °C. The transfer-line, ionization-source, and mass-analyzer temperatures were 280 °C, 230 °C, and 150 °C, respectively. The diluted samples (1% *v*/*v*) were injected in split mode (at a split ratio of 1:15). The analysis running time was ≈ 65 min, and the carrier gas (helium) flow rate was kept constant (1 mL/min).

### 3.3. Gas Chromatography–Flame-Ionization Detector (GC-FID) Analysis of the Volatile Oil

The GC-FID analysis of the *A. absinthium* volatile oil was conducted using a Perkin Elmer Auto System XL instrument that was equipped with a flame-ionization detector (FID). The column used in the oil separation was a ZB-Wax fused silica capillary column (60m × 0.25mm i.d.). The initial temperature of the oven was maintained at 50 °C, and programmed to rise from 50 °C to 220 °C at a rate of 3 °C/min. However, the injector and detector temperatures were 240 °C and 250 °C, respectively. The carrier gas used in the separation was helium, which was passed through the column at a flow rate of 1 mL/min.

### 3.4. Identification of the Volatile Constituents

The volatile oil constituents of the *A. absinthium* were identified by comparing the experimental retention index (RI) of each volatile compound with the retention index obtained for a series of n-alkenes (C_8_ to C_40_) analyzed under similar conditions of experimentation. The reported RI recorded at comparable GC conditions was also used in the compounds identification. In addition, retention time, mass-fragmentation patterns of the peaks, and mass-spectral libraries (NIST-11 and Wiley database) were all used in the identification of the volatile oil constituents of the plant. The areas under the peaks were used to calculate the relative percentages of the constituents.

### 3.5. Antioxidant Activity of A. absinthium Volatile Oil

The antioxidant activity of the *A. absinthium* volatile oil was measured using four in vitro assays as described in the following.

#### 3.5.1. Total Antioxidant Capacity (TAC)

The TAC of the *A. absinthium* volatile oil was evaluated according to the method of Aroua et al. [74] using a molybdate reagent assay. The reagent was formed by dissolving ammonium molybdate ((NH_4_)_2_MoO_4_) in a sodium phosphate buffer (28 mM) to a final concentration of 4 mM, and finally adding H_2_SO_4_ (0.6 M). The working solution was prepared as follows: the diluted *A. absinthium* volatile oil (0.4 mL of methanol containing 200 µg of the volatile oil) was mixed with the molybdate reagent (3.6 mL) in a 20 mL stopped test tube. The tube contents were thoroughly mixed and kept in a 90 °C heated water bath for 30 min. A blank test was also prepared similarly to the previous method, but without involving the oil sample (the oil sample was replaced by 0.4 mL of distilled water). After cooling the mixture to room temperature, the intensity of resulting color was measured at 695 nm. The TAC of the *A. absinthium* volatile oil was calculated as the mean of three replicates equivalent to the well-known antioxidant standard, Trolox.

#### 3.5.2. DPPH Scavenging Activity

The scavenging capability of *A.* *absinthium* volatile to the stable-free radical, DPPH, was measured and calculated as Trolox equivalents [75]. The DPPH working solution was prepared by dissolving 12 mg of the DPPH in 0.1 L of methanol. Then, 1 mL of the *A. absinthium* volatile oil (diluted in a methanol solution and containing 200 µg of the volatile oil) and 1 mL of DPPH working solution was thoroughly mixed and kept at room temperature in a dark place for 30 min before measuring the reduction in the color intensity of the DPPH using a spectrophotometer at 517 nm. The method was repeated three times; methanol was used as a blank, and the scavenging activity of the *A. absinthium* volatile oil was calculated using an equation of the Trolox calibration curve against the DPPH.

#### 3.5.3. Ferric-Reducing Antioxidant Power (FRAP) Assay

The method of Benzie and Strain [76] was used to measure the FRAP activity of the *A. absinthium* volatile oil. The FRAP reagent was freshly prepared using the described method [77]. In test tubes, 0.1 mL of the diluted *A. absinthium* in methanol (a methanol solution containing 200 µg of the oil) was thoroughly mixed with the FRAP reagent (2 mL). The tubes were kept at room temperature for 30 min to develop the reaction color, which was measured at 593 nm. The FRAP activity of the *A. absinthium* volatile oil was calculated as mg Trolox equivalent/g of the volatile oil from three independent experiments using an equation of the FRAP–Trolox calibration curve.

#### 3.5.4. Metal-Chelating-Activity Assay

The iron-chelating activity of the *A. absinthium* volatile oil was measured using the method of Zengin et al. [78]. First, 2 mL of the *A. absinthium* volatile oil diluted in methanol (a methanol solution with 200 µg of the plant’s volatile oil) and 25 µL of FeCl_2_ (2 mM) were thoroughly mixed with ferrozine (100 µL). A blank mixture consisting of all the previous ingredients, but without ferrozine, was prepared in a similar manner. The blank and test mixtures were measured at 562 nm. The chelating activity of the *A. absinthium* volatile oil was calculated using an EDTA calibration curve and expressed as mean of three independent experiments using equivalents to EDTA.

### 3.6. Cytotoxic Assay

The antiproliferative effect of the *A. absinthium* volatile oil was tested against HSF (normal human skin fibroblast cells), A-431 (human epidermoid skin carcinoma), MCF7 (breast adenocarcinoma), and PANC-1 (pancreatic cancer) cell lines, using a standard SRB (sulforhodamine B) assay [70]. Exactly 100 µL of each cell type (at a density of 5 × 10^3^ cells/mL) was seeded in a 96-well microtiter plate in RPMI 1640 medium and incubated for 24 h. Cells were treated with another 100 μL aliquot of the medium containing the *A. absinthium* volatile oil at various concentrations; i.e., 0.01, 0.1, 1, 10, and 100 µg/mL. After 72 h of drug exposure, cells were fixed by replacing the medium with 150 μL of 10% trichloroacetic acid (TCA) and incubated at 4 °C for 1 h. The TCA solution was removed, and the cells were washed 5 times with distilled water. Aliquots of 70 μL SRB solution (0.4% *w*/*v*) were added and incubated in a dark place at room temperature for 10 min. The plates were washed 3 times with 1% acetic acid and allowed to air-dry overnight. Then, 150 μL of TRIS (10mM) was added to dissolve the protein-bound SRB stain; the absorbance was measured at 540 nm using a BMGLA BTECH^®^-FLUO star Omega microplate reader (Ortenberg, Germany). A blank composed of the medium and the *A. absinthium* volatile oil without cells was prepared and treated with the SRB in the same manner, and was used to subtract its value from the absorbance values of the tests. In addition, control (untreated cells) and standard tests (doxorubicin) were prepared for each cell line and similarly treated with the SRB solution. The viability percentages were calculated as follows:

$$\mathrm{Viability}\;\%\;=\left(Absorbance\;of\;\frac{test}{blak}\right)\;\times \;100$$

### 3.7. Statistical Analysis

The results are stated as the mean ± standard error of the mean/standard deviation. A post hoc test was performed using Tukey’s multigroup comparison in GraphPad Prism 8.0.2. The significance value was set at *p* < 0.05.

## 4. Conclusions

In the present study, the volatile oil constituents of *A. absinthium*, the plant species growing in the central area of Saudi Arabia, were isolated and analyzed using GC-MS and GC-FID spectrometric methods. The results of the study revealed distinct variations in the essential-oil constituents of the plant growing in central region of Saudi Arabia and those species of the plant growing in other regions. The higher percentages of davanones (collectively 53%) recorded in the current study represented a clear sign of the environmental effects on the essential-oil constituents of the aromatic plants. The antioxidant and cytotoxic activities of the plant’s essential oil were also evaluated in the current study, and revealed potential health benefits associated with higher antioxidant and lower cytotoxic activity levels obtained for the essential oil of *A. absinthium*. An in vivo antioxidant evaluation of the *A. absinthium* essential oil using animal experimentations, as well as preclinical and clinical trials, are highly recommended for such an important plant.

## Figures and Tables

**Table 1:** Major volatile constituents of *A. absinthium* growing in different locations.

Location	Major Volatile Constituents	Oil Production	Ref.
Saudi Arabia	Camphor, *cis*-davanone, chamazulene, terpinene-4-ol	0.83%	[24,33]
Tunisia and Algeria	𝛽-Thujone, *trans*-sabinene hydrate, 𝛽-selinene, chamazulene, camphor	1.10–1.82% (Tunisia); 0.29% (Algeria)	[26,34,35]
Serbia	Sabinene, 𝛽–thujone, cis-â-epoxyocimene, trans-sabinyl acetate, linalyl 3-methylbutanoate	0.29 to 0.08%	[36]
Lithuania	trans-Sabinyl acetate, β-pinene, trans-thujone, cis-thujone, myrcene	1.2–3.6%	[37,38]
India	Borneol, isobornyl acetate, methyl hinokiate	0.03%	[39]
Tajikistan	Myrcene, cis-chrysanthenyl acetate, a dihydrochamazulene isomer, germacrene D, β-thujone, linalool acetate, α-phellandrene, linalool	0.5–0.8%	[40]
Egypt	a-Phellandrene, terpinen-4-ol		[41]
Ethiopia	Camphor, ethyl (E)-cinnamate, davanone, chamazulene		[27]
Europe	Cineole, myrcene, sabinene, β-thujone, α-thujone, *Epoxyocimene*, sabinyl acetate	0.1–0.9%	[42]
Iran	α-Pinene, sabinene, β-pinene, α-phellandrene, *p*-cymene, chamazulene	0.56–1.05%	[43,44]

**Table 2:** Volatile oil constituents of *A. absinthium* growing in the central area of Saudi Arabia.

No.	Rt (min)	Constituents	RI Exp.	RI Rep.	%
1	15.989	α-Thujene	917	923	0.35
2	17.474	α-Pinene	950	950	1.03
3	17.723	Camphene	956	953	3.27
4	21.151	D-Limonene	1030	1031	0.83
5	21.515	1, 8-Cineole	1038	1038	0.87
6	21.882	β-Ocimene	1046	1043	1.65
7	22.398	α-Terpinene	1056	NF	2.69
8	22.695	cis-Sabinene hydrate	1063	1069	0.55
9	23.262	cis-Linalool oxide	1075	1075	1.55
10	24.586	Linalool	1102	1101	0.57
11	25.113	trans-Thujone	1114	1115	1.41
12	25.614	cis-p-Menth-2-en-1-ol	1124	1124	0.66
13	27.759	Pinocarvone	1170	1168	2.18
14	30.859	Ocimenone	1237	1235	2.03
15	33.576	Bornyl acetate	1297	1289	1.22
16	36.387	Ethyl hydrocinnamate	1351	1347	1.12
17	38.455	α-Gurjunene	1411	1419	7.15
18	40.682	α-Humulene	1465	1455	0.48
19	40.991	Seychellene	1473	1460	0.55
20	41.209	γ-Gurjunene	1478	1475	0.86
21	41.625	γ-Curcumene	1489	1481	1.51
22	41.824	Davana ether *	1493	NF	0.69
23	42.422	α-Muurolene	1508	1508	0.76
24	44.371	Davanone B	1559	1559	0.85
25	44.668	trans-Nerolidol	1565	1564	0.80
26	44.932	Davanone	1573	1570	1.52
27	46.097	cis-Davanone	1603	1594	52.51
28	46.730	Guaiol	1621	1613	0.49
29	46.906	Davanol	1625	1620	0.71
30	47.118	γ-Eudesmol	1631	1630	2.49
31	47.294	Cubenol	1636	1640	1.38
32	48.021	Methyl jasmonate	1656	1652	0.39
33	49.651	Geranyl tiglate	1700	1700	1.48
34	50.750	Chamazulene	1731	1730	3.38
Total concentration of the identified peaks			99.98%		
Nonoxygenated monoterpenes			9.82%		
Oxygenated monoterpenes			11.04%		
Nonoxygenated sesquiterpenes			14.69%		
Oxygenated sesquiterpenes			63.61%		
Cinnamic acid derivatives			1.12%		

* Isomer not identified; RT (min), retention time in minutes; RI exp., experimental retention index; RI rep., reported retention index; NF, not found.

**Table 3:** Antioxidant activity of *A. absinthium* essential oil.

TAC	DPPH-SA	FRAP	MCA
35.59 ± 1.86	10.54 ± 0.31	24.00 ± 0.13	29.87 ± 3.44

The results shown in Table 3 represent data obtained from three independent measurements; means ± SDs were calculated. TAC, total antioxidant capacity in mg Trolox equivalent per gram of the essential oil; FRAP, ferric-reducing antioxidant power in mg Trolox equivalent per gram of the essential oil; DPPH-SA, 2,2-diphenyl-1-picrylhydrazyl-scavenging activity in mg Trolox equivalent per gram of the essential oil; MCA, metal-chelating activity in mg EDTA equivalent per gram of the essential oil.

**Table 4:** Cytotoxic activity of 100 µg/mL *A. absinthium* essential oil and doxorubicin against different tested cell lines.

Cell Lines	MCF-7	Panc-1	A-431	HSF
*A. absinthium* essential oil	Inhibition percentages (%) in cell viability			
	8.27 ± 0.83	12.77 ± 1.11	11.10 ± 0.96	14.56 ± 0.08
DOX	73.05 ± 0.72	81.07 ± 0.36	65.59 ± 0.58	53.31 ± 1.29

## Data Availability

The data presented in this study are available in article and supplementary material.

## Supplementary Materials

The following supporting information can be downloaded at: https://www.mdpi.com/article/10.3390/plants11081028/s1, Figure S1: GC chromatogram of the *A. absinthium* essential oil; Tables S1–S4: Raw data on the cytotoxicity of the *A. absinthium* against MCF-7, Panc-1, A-432, and HSF cell lines, respectively.
