# Supplementary material for: Phytochemical Analysis, Antioxidant Potential, and Cytotoxicity Evaluation of Traditionally Used Artemisia absinthium L. (Wormwood) Growing in the Central Region of Saudi Arabia
Source: Plants (Basel). 2022 Apr 9;11(8):1028. doi: 10.3390/plants11081028 (PMC9029736; doi:10.3390/plants11081028)
Supplement: Supplementary file 1 [file plants-11-01028-s001.zip › plants-1629910-supplementary.pdf]

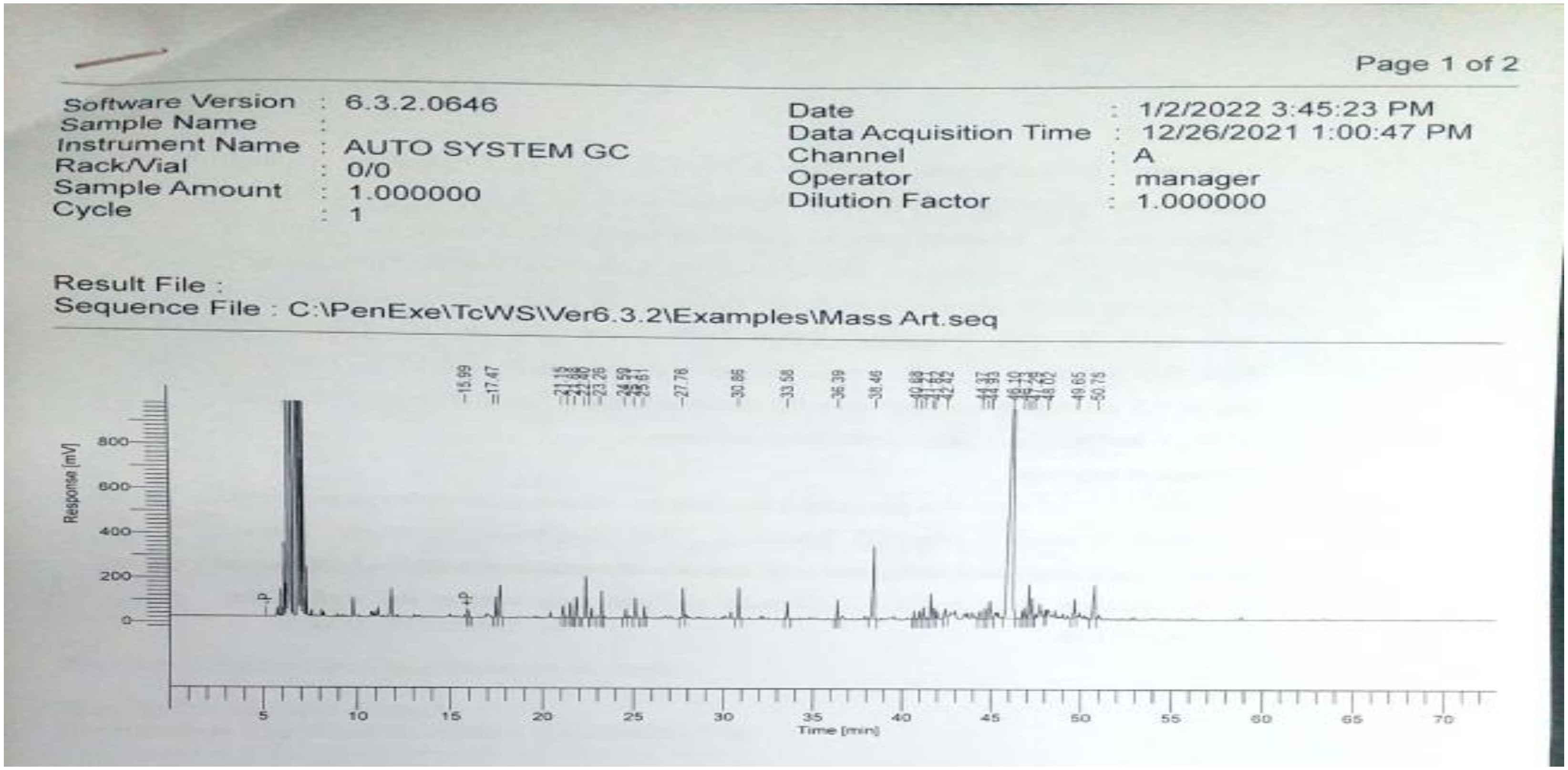

Figure S1: GC-chromatogram of the *A. absinthium* essential oil

Table S1: Raw data of cytotoxicity of *A. absinthium* against MCF-7 cell line

	**Raw data**			Blank Corrected Data			Viability %				
Conc	**1**	**2**	**3**	1	2	3	1	2	3	**Mean**	**STD**
**c**	1.679	1.66	1.693	1.639	1.62	1.653	100	100	100	100	**0**
**0.01**	1.65	1.66	1.651	1.61	1.62	1.611	98.33062	98.94137	98.39169	98.55456	**0.2746486**
**0.1**	1.641	1.644	1.668	1.601	1.604	1.628	97.78094	97.96417	99.42997	98.39169	**0.737971**
**1**	1.633	1.63	1.6301	1.593	1.59	1.5901	97.29235	97.10912	97.11523	97.17223	**0.08497**
**10**	1.619	1.639	1.616	1.579	1.599	1.576	96.4373	97.65879	96.25407	96.78339	**0.6235095**
**100**	1.55	1.523	1.553	1.51	1.483	1.513	92.22313	90.5741	92.40635	91.73453	**0.8239457**
**Blank**	0.04	0.041	0.039	Blank Average		**0.04**	**Control average**		**1.63733**		

Table S2: Raw data of cytotoxicity of *A. absinthium* against Panc-1 cell line

	**Raw data**			Blank Corrected Data			Viability %				
Conc	**1**	**2**	**3**	1	2	3	1	2	3	**Mean**	**STD**
**c**	1.338	1.39	1.393	1.308333	1.360333	1.363333	100	100	100	100	**0**
**0.01**	1.36	1.365	1.37	1.330333	1.335333	1.340333	98.98313	99.35516	99.72718	99.35516	**0.3037562**
**0.1**	1.351	1.352	1.342	1.321333	1.322333	1.312333	98.31349	98.3879	97.64385	98.11508	**0.3345917**
**1**	1.344	1.317	1.312	1.314333	1.287333	1.282333	97.79266	95.78373	95.41171	96.32937	**1.0457921**
**10**	1.261	1.266	1.288	1.231333	1.236333	1.258333	91.61706	91.98909	93.62599	92.41071	**0.8726494**
**100**	1.212	1.213	1.181	1.182333	1.183333	1.151333	87.97123	88.04563	85.66468	87.22718	**1.1052718**
**Blank**	0.029	0.03	0.03	Blank Average		**0.02967**	**Control average**		**1.344**		

Table S3: Raw data of cytotoxicity of *A. absinthium* against A-432 cell line

	**Raw data**			Blank Corrected Data			Viability %				
Conc	**1**	**2**	**3**	1	2	3	1	2	3	**Mean**	**STD**
**c**	2.32	2.312	2.35	2.28	2.272	2.31	100	100	100	100	**0**

**0.01**	2.32	2.3	2.31	2.28	2.26	2.27	99.67939	98.80501	99.2422	99.2422	**0.3569644**
**0.1**	2.288	2.271	2.277	2.248	2.231	2.237	98.28038	97.53716	97.79948	97.87234	**0.3077632**
**1**	2.188	2.144	2.168	2.148	2.104	2.128	93.90848	91.98484	93.0341	92.97581	**0.7864027**
**10**	2.198	2.11	2.21	2.158	2.07	2.17	94.34567	90.4984	94.8703	93.23812	**1.9490823**
**100**	2.042	2.09	2.088	2.002	2.05	2.048	87.5255	89.62402	89.53658	88.89537	**0.9692969**
**Blank**	0.039	0.04	0.041	Blank Average		**0.04**	**Control average**		**2.28733**		

Table S4: Raw data of cytotoxicity of *A. absinthium* against HSF cell line

	**Raw data**			Blank Corrected Data			Viability %				
Conc	**1**	**2**	**3**	1	2	3	1	2	3	**Mean**	**STD**
**c**	2.819	2.86	2.889	2.779	2.82	2.849	100	100	100	100	**0**
**0.01**	2.75	2.756	2.818	2.71	2.716	2.778	96.2358	96.44886	98.65057	97.11174	**1.0915854**
**0.1**	2.675	2.722	2.728	2.635	2.682	2.688	93.57244	95.24148	95.45455	94.75616	**0.8415186**
**1**	2.689	2.616	2.67	2.649	2.576	2.63	94.0696	91.47727	93.39489	92.98059	**1.0981123**
**10**	2.597	2.617	2.502	2.557	2.577	2.462	90.80256	91.51278	87.42898	89.91477	**1.7814769**
**100**	2.447	2.448	2.443	2.407	2.408	2.403	85.47585	85.51136	85.33381	85.44034	**0.0767133**
**Blank**	0.039	0.041	0.04	Blank Average		**0.04**	**Control average**		**2.816**		
